# Coping Strategies Moderate the Association Between Food Insecurity and Food Consumption Among Lebanese Adults

**DOI:** 10.1002/puh2.70224

**Published:** 2026-04-07

**Authors:** Souheil Hallit, Rawan Achhab, Krystel Kfoury, Yonna Sacre

**Affiliations:** ^1^ School of Medicine and Medical Sciences Holy Spirit University of Kaslik Jounieh Lebanon; ^2^ Applied Science Research Center Applied Science Private University Amman Jordan; ^3^ Department of Nutrition and Food Sciences Faculty of Arts and Sciences Holy Spirit University of Kaslik Jounieh Lebanon

**Keywords:** adults, coping strategies, food consumption, food insecurity, Lebanon

## Abstract

**Background:**

The prevalence of food insecurity (FI) is increasing worldwide, leading to several health issues. The impact of FI on adverse health outcomes, especially on food consumption, may be partly influenced by the strategies that household members use to cope with limited food access. This study sought to examine the moderating effect of coping strategies on the relationship between FI and food consumption among Lebanese adults.

**Methods:**

A total of 385 Lebanese adults participated in this cross‐sectional study. FI and food consumption were evaluated using the Household Food Insecurity Access Scale and the Food Consumption Score, respectively. Coping strategies were measured using the Coping Strategy Index.

**Results:**

The moderation analysis results showed that FI was not significantly associated with food consumption (*p* = 0.499). However, higher coping strategy scores were significantly associated with higher food consumption (beta = 0.45; *p* < 0.001). Moreover, coping strategies moderated the association between FI and food consumption (beta = −0.02, *t* = −2.33, *p* = 0.020, 95% CI −0.04; −0.003). At low, moderate, and high levels of coping strategies, greater FI was significantly associated with lower food consumption.

**Conclusion:**

Our results underscore the complex interplay between FI and coping strategies, highlighting the importance of targeted interventions that strengthen adaptive coping mechanisms to mitigate the adverse effects of FI on nutritional well‐being. Future research should identify the specific coping strategies individuals adopt in response to FI and examine their long‐term effects on food consumption through longitudinal studies. Policy initiatives and intervention programs that integrate food security measures with coping strategy support may help alleviate the negative consequences of FI.

## Abstract 2

The current findings indicate that the PDSS is an effective and valid instrument for measuring daytime sleepiness among Arabic‐speaking adolescents. The PDSS in the Arabic version allows clinicians and practitioners to evaluate daytime sleepiness among younger populations and take action to mitigate the worsening of such issues, which could increase in severity overtime.

## 1 Introduction

Food consumption encompasses the social and ceremonial practices surrounding eating, including language, gestures, preparation, selection, and ordering of food [1]. Maintaining healthy food consumption is fundamental for overall health and nutrition, as it helps to protect individuals against various chronic noncommunicable diseases, such as cancer, diabetes, and cardiovascular diseases. A balanced diet involves consuming a wide variety of foods while limiting the intake of sugar, salt, and industrially produced trans fats [2, 3]. One of the factors that can seriously affect food intake frequency, dietary diversity, and nutritional adequacy is food insecurity (FI).

### 1.1 The Relationship Between FI and Food Consumption

FI refers to a household‐level social and economic condition of uncertain or limited access to safe, sufficient, and nutritious food that meets one's food preferences and dietary needs for a healthy and active life [4]. FI represents a multidimensional construct encompassing availability, accessibility, utilization, and stability of food [5]. In other terms, FI extends broader than just the food consumption behavior to include food stability and access over time. FI can substantially influence dietary behaviors and patterns, often leading to poorer diet quality, reduced dietary diversity, and maladaptive eating practices [6]. Inadequate access to food can have both immediate and long‐term effects on nutritional status and dietary behaviors [7]. This condition often forces individuals to skip meals and consume low‐quality foods, such as sugar‐sweetened beverages, refined grains, confectioneries, and snacks rich in saturated and trans fats, which can contribute to the development of chronic diseases, including diabetes, hypertension, obesity, and certain types of cancer [8]. A systematic review showed that food‐insecure individuals were more likely to report higher intakes of unhealthy foods (e.g., added sugars, sugar‐sweetened beverages, and fast foods), lower intakes of healthy foods (e.g., vegetables, fruits, and whole grains), as well as less frequent consumption of breakfast and evening meals [9]. Although FI is a well‐established predictor of poorer dietary outcomes, the mechanisms underlying this relationship are under‐researched and largely unknown. The present study proposes to investigate the conditions under which FI relates to food consumption by examining coping strategies as a moderator of this relationship. Testing the moderating role of coping strategies may contribute to deeper insights and a more nuanced understanding of the interplay between these constructs beyond main effects of FI on food consumption alone.

### 1.2 The Moderating Role of Coping Strategies

Coping strategies generally involve accepting or tolerating adverse circumstances and realities while striving to maintain psychological stability and a healthy sense of self [10]. To manage FI, households often adopt a variety of adaptive behaviors, including purchasing less expensive foods, borrowing food or money, taking out loans, relying on family or friends for support, reducing portion sizes, or even resorting to begging for food [11]. From a theoretical perspective, coping strategies are not merely behavioral responses but constitute a managed process through which households negotiate insecure access to food. Hadley and Crooks conceptualize FI as a dynamic, biosocial process in which households deploy context‐specific coping responses that modify health outcomes [12]. In their framework, FI triggers a range of food‐based and non‐food‐based coping mechanisms, which, in turn, shape nutritional status, chronic disease risk, and overall well‐being. Importantly, these coping responses may buffer adverse outcomes in the short term, whereas others may compromise long‐term dietary adequacy or health. This perspective aligns with stress‐coping theory [13], which hypothesizes that individual and household responses to stressors can alter the magnitude and direction of health‐related outcomes. In the context of FI, coping strategies may therefore function as modifiers of how FI translates into changes in food consumption.

### 1.3 The Lebanese Context

Internationally, FI has emerged as a significant public health concern and is projected to worsen in both high‐income [14] and low‐income countries [15]. Following the COVID‐19 pandemic, the prevalence of FI increased by 27% compared to pre‐pandemic levels [16]. Similar to other Arab and Mediterranean countries, a 2015 cross‐sectional study found that 40% of Lebanese residents in Beirut were food insecure and exhibited poorer dietary quality, higher rates of overweight, and greater physical inactivity compared to food‐secure individuals [17]. Over the past several years, Lebanon has faced a profound economic and financial collapse triggered by the abrupt imposition of capital controls in October 2019, which led to the emergence of a parallel exchange market, rapid currency depreciation, and sharp rises in consumer prices [18]. Since then, the Lebanese Lira has lost the vast majority of its value relative to the US dollar, and Lebanon has experienced one of the highest inflationary crises in recent history. According to the Central Administration of Statistics, annual inflation reached upwards to 200% in the early 2020s before moderating to approximately 14.8% in 2025 [19]. The decline in inflation rates over time masks the fact that overall price levels remain greatly elevated compared to precrisis norms, contributing to widespread increases in the cost of food, decline of household purchasing power, and higher rates of FI. These developments have negatively affected people's purchasing power and dietary choices, leading to increased consumption of processed foods, which are generally more affordable and have longer shelf lives than nutrient‐dense, perishable alternatives [16]. Globally—and particularly in Lebanon—poor food security and inadequate food consumption have significantly influenced individuals’ eating patterns, health, and overall quality of life [15, 20]. Consequently, many have adopted various coping mechanisms in response to the environmental and economic challenges that have intensified in recent years [21].

### 1.4 Rationale and Objectives of the Current Study

Although existing literature has extensively documented the association between FI and adverse dietary outcomes, less attention has been given to the potential role of coping strategies in shaping this relationship. From a theoretical perspective, coping mechanisms may act as moderating factors, influencing the strength or direction of the relationship between FI and food consumption. For instance, individuals who adopt more adaptive coping strategies may buffer the negative effects of FI on dietary behaviors, whereas maladaptive coping responses may exacerbate poor consumption patterns. Thus, a key gap that remains is to understand whether coping strategies merely accompany FI or whether they modify how FI translates into actual dietary behaviors. Given the profound economic crisis in Lebanon and the widespread adoption of coping mechanisms to manage resource constraints [22], examining the moderating role of coping strategies may provide valuable insight into how FI translates into dietary outcomes within this context.

Therefore, the objective of the present study was to examine the moderating effect of coping strategies on the relationship between FI and food consumption among a sample of Lebanese adults. The moderation analysis will be adjusted for demographic (age, gender) and biometric (e.g., body mass index, physical activity) factors, given their established associations with food consumption, to ensure that interaction estimates are not biased by these background characteristics. Indeed, previous findings showed that physical activity may serve as a justification cue that alters eating behavior, particularly by increasing food intake under certain psychological conditions [23]. This suggests that engagement in physical activity may independently affect food consumption patterns, regardless of FI status. Building on the biosocial coping framework proposed by Hadley and Crooks [12], we hypothesized that—after controlling for covariates—coping strategies would moderate the association between FI and food consumption, such that individuals relying on more coping strategies in response to food scarcity would show a weaker negative association between FI and food consumption compared to those having less coping behaviors.

## 2 Materials and Methods

### 2.1 Study Design, Participants, and Sample Size

A cross‐sectional study was conducted between August 2023 and January 2024. A total of 385 Lebanese adults voluntarily participated. Participants were recruited through an online Google Forms link disseminated via social media platforms (WhatsApp, Instagram, and Messenger) using a snowball sampling technique. The research team initially invited acquaintances to participate, and respondents were asked to forward the link to other people who met the inclusion criteria. Eligible participants were Lebanese residents aged 18 years or older. Participants were asked via a self‐reported screening question whether they had any chronic illness affecting food intake; individuals who responded affirmatively were excluded from participation in the survey. Informed consent was obtained electronically prior to completing the anonymous, self‐administered questionnaire, which required around 10 min to complete.

Anthropometric and body composition measurements were carried out in person; weight, height, waist circumference, and body composition were measured after meeting participants face‐to‐face in meeting centers. Waist circumference was measured using a waist tape; body composition was determined using a validated bioelectrical impedance analysis device under standardized settings; and height and weight were measured using calibrated and digital equipment.

#### 2.1.1 Minimum Sample Size

According to the G*Power software (multiple regression *R* ^2^ deviation from zero), the minimum required sample size (*n*) to have enough statistical power was estimated at 295 participants, based on a 5% effect size, 5% type 1 error, 80% power, and 7 predictors to be entered in the multivariable model.

##### 2.1.1.1 Questionnaire

A structured questionnaire comprising validated instruments was used to meet the study objectives. The first section gathered sociodemographic data and information related to FI, food quality, body composition, anthropometric characteristics, and physical activity. The second part included the following scales.

##### 2.1.1.2 Food Insecurity

The Arabic version of the Household Food Insecurity Access Scale (HFIAS) [24] was used. It consists of nine items scored from 0 (never) to 3 (often), yielding a total score between 0 and 27. On the basis of the total score, participants were classified as food secure (0), mildly food insecure (1–3), moderately food insecure (4–10), and severely food insecure (11–27) [24].

##### 2.1.1.3 Food Consumption

The FCS, developed by the World Food Programme, was used to assess household dietary diversity and food frequency. Participants were asked to report the number of days (0–7) each predefined food group was consumed during the previous 7 days. The following eight food groups were included: staples (cereals and tubers), pulses, vegetables, fruits, meat and fish, milk and dairy products, sugar, and oil. Each food group was assigned a standardized weight reflecting its relative nutritional value (staples = 2, pulses = 3, vegetables = 1, fruits = 1, meat/fish = 4, milk = 4, sugar = 0.5, and oil = 0.5). The FCS was calculated by multiplying the frequency of consumption of each food group by its corresponding weight and summing the results.

##### 2.1.1.4 Physical Activity

The International Physical Activity Questionnaire‐Short Form (IPAQ‐SF) [25], validated in Arabic [26], was employed to measure levels of physical activity, including walking, moderate‐intensity, and vigorous‐intensity exercise. Activity levels were categorized as low (no or minimal activity), moderate (≥3 days of vigorous activity defined as ≥20 min per day or 5 or more days of moderate activity or walking for at least 30 min per day, or 5 or more days of any combination of walking, moderate, or vigorous activities), and high (≥3 days of vigorous activity or ≥7 days of combined activities [walking, moderate, or vigorous activities]) [25].

##### 2.1.1.5 Coping Strategies

The Coping Strategy Index (CSI) [27] was used to assess the frequency and severity of coping behaviors in response to food scarcity. Nine items measured behaviors, such as consuming less preferred foods, borrowing foods, buying food on credit, limiting portion sizes at meal times, going an entire day without eating, and sending household members to beg. Each response was multiplied by a severity weight: 1 (low), 2 (medium), and 3 (severe). Higher scores would reflect higher coping strategies [27].

For anthropometric measurements, weight (kg) was measured using an electronic scale placed on a flat surface and height (cm) using a stadiometer. Body composition was assessed using a bioelectrical impedance device (Bodecoder Body Composition Analyzer), a unique mono‐frequency BIA body composition scale that provides accurate, inexpensive, rapid, and noninvasive measurement of fat mass and fat‐free mass.

### 2.2 Data Analyses

No missing data were found in the database because all questions were required in the Google Forms. The SPSS software v.25 was used for the statistical analysis. The food consumption score was normally distributed since the skewness (=0.532) and kurtosis (=−0.259) values were inside the −1 and +1 interval [28]. The student *t* test was used to compare two means and the Pearson test to correlate two continuous variables. Moderation analysis was conducted using ordinary least squares (OLS) in PROCESS Macro version 4.2 model 1. Continuous predictors (FI and coping strategies) were mean‐centered prior to computing the interaction term to reduce multicollinearity. The interaction term was computed as the product of the centered FI and coping strategies variables. In this model, the main effect of FI represents its association with food consumption when coping strategies are at their mean level and vice versa. Conditional effects of FI on food consumption were examined at low (−1 SD), moderate (mean), and high (+1 SD) levels of coping strategies. The model was adjusted for variables with *p* < 0.25 in the bivariate analysis. This threshold was selected in accordance with methodological recommendations [29] suggesting a more liberal criterion at the bivariate stage reduces the risk of excluding potential confounders that may become significant in multivariable models. Multicollinearity was assessed using variance inflation factors (VIF) and tolerance statistics. All values indicated no evidence of problematic multicollinearity. *p* < 0.05 was deemed statistically significant.

## 3 Results

### 3.1 Sociodemographic and Other Characteristics of the Study Sample

Three hundred and eighty‐five participants participated in this study, with the majority aging between 35 and 49 years (39.0%) and 59.0% females. Other descriptive statistics of the sample can be found in Table 1.

**TABLE 1:** Sociodemographic and other characteristics of the sample (*N* = 385).

Variable	*N* (%)
Age (years)	
20–34	122 (31.7)
35–49	150 (39.0)
50–64	113 (29.4)
Gender	
Male	158 (41.0)
Female	227 (59.0)
Mean ± SD	
Body mass index (kg/m^2^)	26.39 ± 5.91
Waist circumference (cm)	83.74 ± 9.50
Body fat percentage	28.77 ± 11.43
Free fat mass	71.26 ± 11.45
Food consumption	45.07 ± 20.62
Food insecurity	12.89 ± 6.47
Coping strategies	66.24 ± 19.79

*Note:* kg/m^2^, kilogram per square meter; cm, centimeter.

Abbreviation: SD, standard deviation.

### 3.2 Bivariate Analysis of Factors Associated With Food Consumption

A higher mean food consumption score was found in participants who have a low physical activity level (Table 2). Moreover, the results showed that higher coping strategies and higher waist circumference were significantly associated with higher food consumption, whereas higher FI was significantly associated with lower food consumption (Table 3).

**TABLE 2:** Bivariate analysis of factors associated with food consumption.

Variable	Mean ± SD	*t*/*F*	df/df1, df2	*p*
Age (years)		2.44	2, 382	0.089
20–34	44.81 ± 16.75			
35–49	47.62 ± 21.30			
50–64	41.98 ± 23.09			
Gender		−0.98	383	0.327
Male	43.89 ± 17.82			
Female	45.90 ± 22.36			
Physical activity		9.68	2, 382	**<0.001**
Low	49.29 ± 22.85			
Moderate	46.17 ± 19.55			
High	38.55 ± 16.75			

*Note:* Numbers in bold indicate significant *p* values <0.05.

Abbreviations: df, degree of freedom; *p*, *p* value; SD, standard deviation.

**TABLE 3:** Correlations of continuous variables with food consumption.

	1	2	3	4	5	6
1. Food consumption	1					
2. Food insecurity	−0.26***	1				
3. Coping strategies	0.18**	0.17***	1			
4. Body mass index	0.10	0.11*	0.14**	1		
5. Waist circumference	0.15**	0.03	0.04	0.78***	1	
6. Body fat	0.06	0.07	0.08	0.80***	0.66***	1
7. Free fat mass	−0.06	−0.07	−0.08	−0.79***	−0.65***	−0.99***

*Note:* Numbers in the table refer to Pearson correlation coefficients.

*p < 0.05.

**p < 0.01.

***p < 0.001.

### 3.3 Moderation Analysis

**FIGURE 1:**
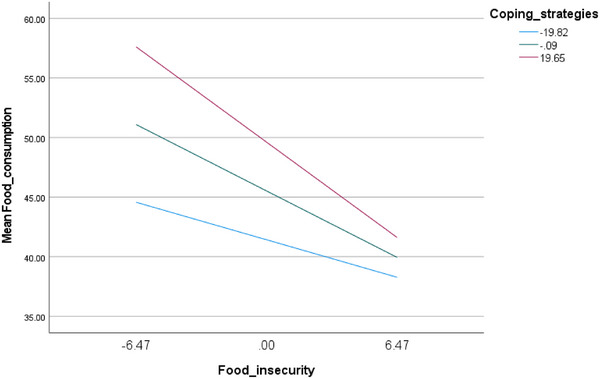
Interaction of coping strategies between food insecurity and food consumption.

The moderation analysis was adjusted over the following variables: physical activity, age, BMI, and waist circumference. The results showed that higher coping strategies were associated with higher food consumption, whereas higher FI was significantly associated with lower food consumption. Moreover, coping strategies moderated the association between FI and food consumption (beta = −0.02, *t* = −2.37, *p* = 0.018, 95% CI −0.04; −0.003) (Table 4). At low, moderate, and high levels of coping strategies, higher FI was significantly associated with less food consumption, but the magnitude of reduction increases at higher coping levels (Table 5, Figure 1).

**TABLE 4:** Moderating effects of coping strategies between food insecurity and food consumption.

	Beta	*t*	*p*	95% CI
Food insecurity	−0.86	−5.49	**<0.001**	−1.17; −0.55
Coping strategies	0.21	3.98	**<0.001**	0.11; 0.31
Interaction food insecurity by coping strategies	−0.02	−2.37	**0.018**	−0.04; −0.003

*Note:* Numbers in bold indicate significant *p* values.

**TABLE 5:** Conditional effects of the focal predictor (food insecurity) at values of the moderator (coping strategies).

Levels of coping strategies	Beta	*t*	*p*	95% CI
Low (=−19.82)	−0.49	−2.13	**0.034**	−0.94; −0.04
Moderate (=−0.09)	−0.86	−5.47	**<0.001**	−1.17; −0.55
High (=19.65)	−1.23	−5.67	**<0.001**	−1.66; −0.81

*Note:* Numbers in bold indicate significant *p* values.

## 4 Discussion

Our findings suggest that higher coping strategies were associated with higher food consumption and that at low, moderate, and high levels of coping strategies, higher FI was significantly associated with less food consumption.

Regarding the association between FI and food consumption, the results indicated that higher FI was significantly associated with lower food consumption. This finding is consistent with several previous studies suggesting that FI is linked to poor dietary quality in various populations [30, 31, 32]. For instance, a study among lactating and non‐lactating mothers with children under 2 years—a physiologically vulnerable group—reported that approximately 13.1% of those experiencing mild to moderate FI showed a significant reduction in the number of meals, snacks, and specific food types consumed [33]. The latter study supports the close connection between FI and food consumption among a sample with heightened nutritional needs and is in line with our findings involving a general population sample with wider physiological and socioeconomic conditions. These results reinforce the broader evidence that constrained food access can directly translate into reduced dietary intake across different demographic contexts. Food consumption patterns in Lebanon may differ from those in other countries due to cultural dietary habits, variations in food availability, and the presence of strong social support systems. In the context of Lebanon's prolonged economic crisis and high inflation, households may be compelled not only to modify food types based on affordability but also to reduce overall food consumption, particularly when coping resources are limited. Future studies should therefore focus on assessing food quality and dietary diversity rather than solely evaluating total food consumption.

With respect to the correlation between coping strategies and food consumption, our findings revealed that higher coping strategy scores were associated with higher food consumption. This finding suggests that coping strategies may function as adaptive resource‐mobilization mechanisms rather than solely as deprivation responses. Recent literature conceptualizes coping not only as a marker of vulnerability but also as an active behavioral process through which households reorganize resources, access social support, and protect dietary intake under economic strain [34]. This result contrasts with a 2020 study showing that coping strategies were more prevalent among low‐income, less educated, and self‐employed individuals, leading to the consumption of less expensive, less preferred foods, and increased meal rationing [35]. Similarly, in a study conducted in Sudan, Abdalla et al. found that gathering wild food and consuming seed stocks reserved for the next planting season were practiced weekly to secure food consumption, whereas rationing strategies, such as skipping meals or going an entire day without eating, were used twice a week to reduce food costs—resulting in diets lower in nutritional value but higher in fat and sugars [36].

It is important to acknowledge that coping strategies may contribute to reduced food intake, particularly through behaviors, such as meal rationing, portion reduction, and prolonged fasting, which are commonly used by food‐insecure individuals when food access becomes severely limited [37]. Such strategies can lead to malnutrition, weight loss, and other adverse health outcomes [38]. Moreover, individuals facing severe FI often prioritize feeding vulnerable household members, such as children, whereas adults consume less or skip meals entirely [39].

In the Lebanese context, however, coping strategies may take a distinct form. Individuals often depend on strong family and community support networks [40], as well as food aid and remittances from abroad, which may enable households to maintain or even increase food consumption despite financial hardship. In our sample, coping mechanisms, such as borrowing food or money, relying on relatives, or substituting cheaper but more abundant staples, may have helped sustain overall food intake. This association underscores the dual nature of coping mechanisms: Although some strategies may support sustained food intake, others can lead to reductions in both the quantity and quality of meals.

The moderation analysis revealed that FI and coping strategies together significantly predicted food consumption, with higher levels of FI associated with lower food consumption across all levels of coping strategies. Globally, approximately 2.4 billion people experience moderate FI, and 900 million face severe FI due to factors, such as climate change, conflict, inequality, discrimination, and weak government and health systems [41]. To adapt to these challenges, various coping mechanisms are employed to different extents, with reliance on less preferred and less expensive foods being the most common. However, such strategies often result in reduced consumption of healthy, nutrient‐dense foods.

The present findings suggest that higher FI is significantly associated with lower food consumption and that increased reliance on coping strategies is also linked to reduced intake of nutrient‐rich foods, contributing to poor diet quality and inadequate vitamin and mineral intake. On the basis of these results, it can be hypothesized that certain coping mechanisms—such as eating elsewhere, begging for food, or skipping meals—contribute to both reduced food consumption and poorer dietary quality. The impact of FI on food consumption was, therefore, moderated by several coping behaviors.

A 2017 study in Bangladesh demonstrated that food‐insecure households adopted higher levels of coping strategies, which in turn led to poorer food consumption. Specifically, food‐compromising strategies related to food quality and quantity were 4.54 times higher among households with severe FI but 0.3 times lower among those with moderate FI [10]. These findings reinforce the conclusion that coping strategies play a moderating role in the relationship between FI and food consumption. It is plausible that food‐insecure individuals without coping mechanisms have a very limited and poor‐quality diet, whereas those adopting coping mechanisms may exercise greater control over food type and quality.

### 4.1 Limitations

Several unmeasured confounding variables may have influenced the results of this study, including household size, food quality, dietary diversity, and mental health status. Furthermore, the cross‐sectional design limits the ability to establish causal relationships between food security, food quality, and coping strategies. The use of a convenient sampling technique also restricts the generalizability of the findings to the broader Lebanese population. Potential information bias may have arisen from participants’ misinterpretation or misunderstanding of certain questions. Residual confounding bias may be present, as some factors (i.e., education level, income, employment status, marital status, and household size) were not included in the survey. The impact of FI on food consumption may vary according to educational level. For instance, even among food‐insecure individuals, those with higher education may possess greater nutritional awareness and prioritize healthier choices. Highly educated individuals in urban areas may also be more inclined to regulate their food intake by emphasizing fruits, vegetables, and lean meats while limiting foods high in fats and sugars.

In addition, FI status was assessed through self‐report measures, which may have resulted in misclassification bias, particularly if participants under‐ or over‐reported their level of food security. Anthropometric indicators (BMI and waist circumference) were self‐reported, which may introduce measurement error due to recall or social desirability bias. Although multicollinearity in the final adjusted model showed acceptable VIF values, the inclusion of correlated anthropometric variables may still introduce residual overlap in variance that could influence parameter stability.

## 5 Conclusion

The results of this study revealed that coping strategies moderated the association between FI and food consumption, with higher levels of FI being associated with lower food consumption, low, moderate, and high levels of coping strategies. These findings highlight the complex interplay between FI and coping behaviors, emphasizing the importance of developing targeted interventions that strengthen adaptive coping mechanisms to mitigate the adverse effects of FI on nutritional well‐being.

Future research should investigate the specific coping strategies individuals adopt in response to FI and evaluate their long‐term impacts on food consumption through longitudinal or cohort studies. Moreover, intervention‐based programs and policy initiatives that integrate food security measures with coping strategy support could play a crucial role in improving dietary outcomes, enhancing community resilience, and reducing the negative impact of FI.

## Author Contributions

Yonna Sacre designed the study. Rawan Achhab collected the data and wrote the manuscript. Souheil Hallit carried out the analysis and interpreted the results. Souheil Hallit, Krystel Kfoury, and Yonna Sacre reviewed the article for intellectual content. All authors reviewed the final manuscript and gave their consent.

## Funding

The authors have nothing to report.

## Ethics Statement

The research protocol was approved by the ethics committee of the Holy Spirit University of Kaslik. The study was performed following the standards for medical research involving human subjects recommended by the Declaration of Helsinki for human research.

## Consent

All participants were provided full information on the study and provided their informed consent to participate.

## Conflicts of Interest

The authors declare no conflicts of interest.

## Data Availability

The datasets generated and/or analyzed during the current study are not publicly available but are available from the corresponding author on reasonable request.
